# Supplementary material for: Effectiveness of an O-Alkyl Hydroxamate in Dogs with Naturally Acquired Canine Leishmaniosis: An Exploratory Clinical Trial
Source: Animals (Basel). 2022 Oct 7;12(19):2700. doi: 10.3390/ani12192700 (PMC9559384; doi:10.3390/ani12192700)

Supplementary Table S1. Evolution of the parasite loads and the clinical Score throughout the experiment in dogs treated with meglumine antimoniate. A value "-" for parasite load indicates a negative result in qPCR. BM = Bone Marrow parasite load, indicated in parasites/µL; LN = Lymph Node parasite load, indicated in parasites per microgram of DNA; PB = Peripheral Blood parasite load, indicated in parasites per 100 µL; CS = Clinical Score.

		Parasite load or clinical score			
dog	tissue	day 0	day 60	day 120	day 210
1	BM	-	0.01	-	-
	LN	11.2	1.6	-	17.8
	PB	0.33	-	1.1	-
	CS	3	6	4	1
2	BM	-	-	-	-
	LN	172.1	1.1	-	-
	PB	0.23	0.19	26.2	-
	CS	2	1	2	1
3	BM	2124.4	1158.4	185.4	1.8
	LN	63.6	66.2	129.2	69.6
	PB	105.9	48.2	1.0	-
	CS	18	24	18	20
4	BM	0.01	0.01	0.02	0.13
	LN	-	-	7.1	5.8
	PB	0.68	-	0.42	-
	CS	2	3	2	2
5	BM	0.01	0.02	0.06	0.09
	LN	1.3	25.4	108.4	26.7

	PB	0.10	0.34	1.9	0.67
	CS	2	7	5	5
6	BM	0.29	0.11	-	-
	LN	-	-	-	0.04
	PB	1.50	-	3.4	0.29
	CS	2	2	1	4

Supplementary Table S2. Evolution of the antibody titres and the cytokine expression levels throughout the experiment in dogs treated with meglumine antimoniate. Value = 0 for antibody titre denotes an antibody titre lower than 20. IgG = Total IgG antibody titre; IgG1 = IgG1 antibody titre; IgG2 = IgG2 antibody titre; IFNG = Interferon gamma relative expression level; IL4 = Interleukin 4 relative expression level.

		Antibody titre or cytokine expression level			
Dog	Parameter	day 0	day 60	day 120	day 210
1	IgG	640	320	320	320
	IgG1	40	40	40	80
	IgG2	1280	640	1280	640
	IFNG	4.7	2.0	12.0	0.25
	IL4	0.16	0.41	0.13	0.03
2	IgG	40	80	80	40
	IgG1	0	0	0	0
	IgG2	20	20	0	0
	IFNG	5.7	4.3	3.3	10.6
	IL4	3.6	0.79	1.3	0.11
3	IgG	1280	1280	1280	1280

	IgG1	1280	1280	1280	1280
	IgG2	1280	1280	1280	1280
	IFNG	3.4	0.02	0.36	0.10
	IL4	0.03	0.03	0.03	0.03
4	IgG	80	80	160	320
	IgG1	0	20	0	80
	IgG2	160	80	20	640
	IFNG	0.27	0.04	0.04	0.12
	IL4	0.11	0.04	0.75	0.21
5	IgG	320	80	80	40
	IgG1	0	40	0	0
	IgG2	160	320	80	80
	IFNG	1.0	0.46	7.1	0.10
	IL4	0.03	0.01	1.1	32.7
6	IgG	40	20	0f	20
	IgG1	0	0	0	0
	IgG2	40	40	0	40
	IFNG	0.13	0.95	11.7	3.1
	IL4	3.6	0.09	0.11	0.11

Supplementary Table S3. Baseline clinical picture of the animals before the intervention: breed, age, sex and estimated time from infection. Age is shown in years. Estimated time of infection is shown in months according to the owners' assessment. F =Female, M =Male, WL =Weight loss, LA =Lymphadenomegaly, ED =Exfoliative dermatitis, OG =Onychogryphosis, ME =Mentation, UK =Uveitis or Keratitis, HK =hyperkeratosis in truffle or pads, AL =alopecia, AA =Arregenerativeanemia, AG =Low albumin/globulin ratio, UD =Ulcerative dermatitis, ND =Nodular dermatitis.

Dog	Breed	Age	Sex	Time from infection	Clinical signs	IFAT titre
1	Labrador	3	F	6	WL, LA, ED	1280
2	Mongrel	3	M	6	LA, OG	160
3	Boxer	4	M	9	WL, ME, LA, UD, OG, HK, AL, AA, AG, ED	1280
4	Malinois shepherd	5	F	8	LA, OG, ME	320
5	Boxer	3	M	7	LA, UK	160
6	Hound	3	M	6	WL, LA	80
7	Mastiff	1	M	2	WL, LA, UD, ME	80
8	Mongrel	5	M	9	LA, ED	640
9	Mongrel	3	F	4	LA, OG, UK, HK	320
10	Labrador	3	F	7	LA, OG	80
11	Hound	3	M	7	LA, OG, UK, WL, ME, ED	80
12	Hound	3	M	8	OG, LA, UK, WL, ED, HK	320
13	Mongrel	3	F	6	LA, OG, ED, UD, ND,	1280

					WL	
14	Shar Pei	2	M	4	UD, UK	160
15	Labrador	2	M	4	WL, UD, LA	80
16	German Shepherd	6	M	3	LA, ED	80
17	Hound	4	M	3	LA, OG, UD, WL	320
18	Duck trolling retriever	2	M	1	UD, ED	80

Supplementary Figure S1. Evolution of parasite load in bone marrow. The figure shows the average percentage (%) parasite load compared to the baseline parasite load before treatment (on day 0). Groups treated with MTC-305, meglumine antimoniate (MA) or a combination of both (COMB).

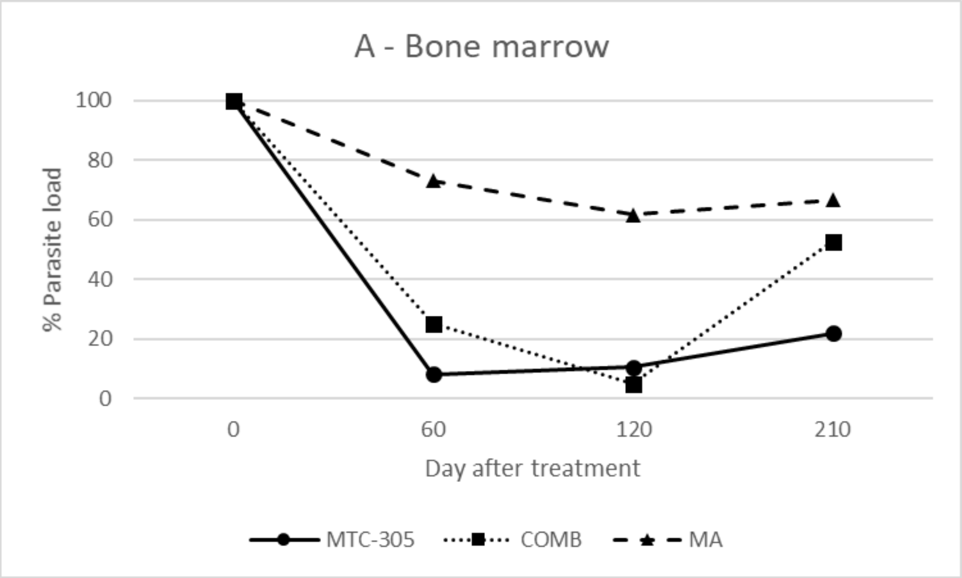

Supplementary Figure S2. Evolution of parasite load in lymph node. The figure shows the average percentage (%) parasite load compared to the baseline parasite load before treatment (on day 0). Groups treated with MTC-305, meglumine antimoniate (MA) or a combination of both (COMB).

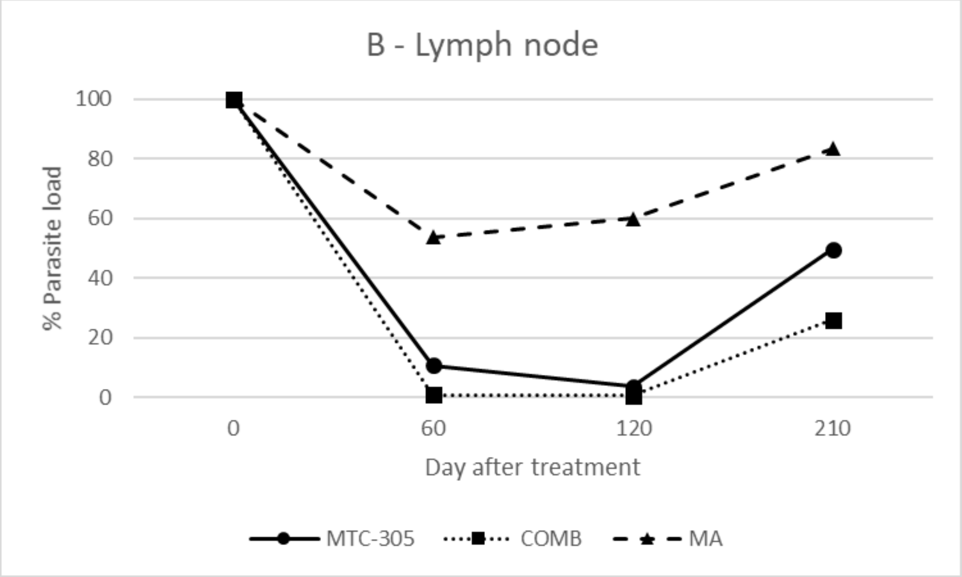

Supplementary Figure S3. Evolution of parasite load in peripheral blood. The figure shows the average percentage (%) parasite load compared to the baseline parasite load before treatment (on day 0). Groups treated with MTC-305, meglumine antimoniate (MA) or a combination of both (COMB).

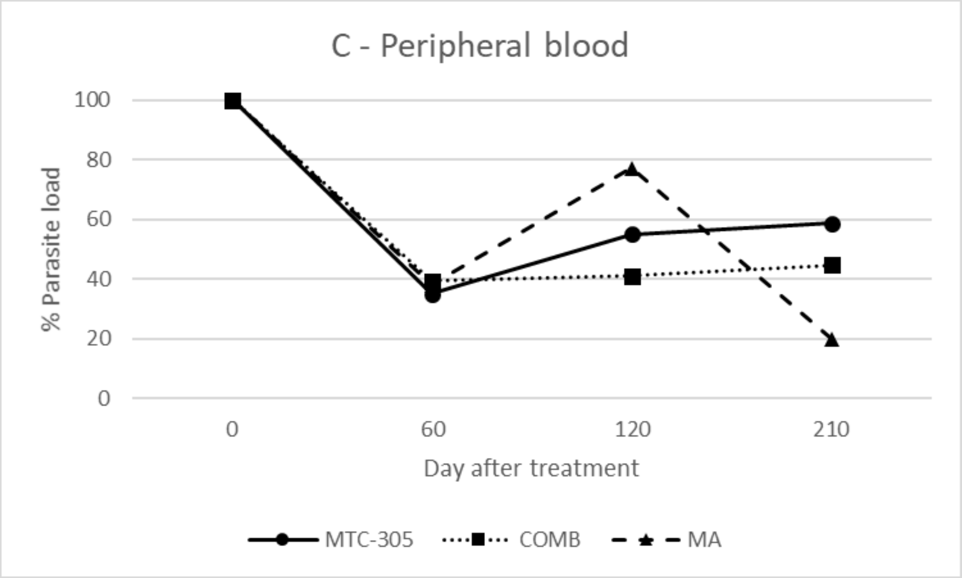

Supplementary Figure S4. Evolution of clinical score. The figure shows the average percentage (%) clinical score compared to the baseline before treatment (on day 0). Groups treated with MTC-305, meglumine antimoniate (MA) or a combination of both (COMB).

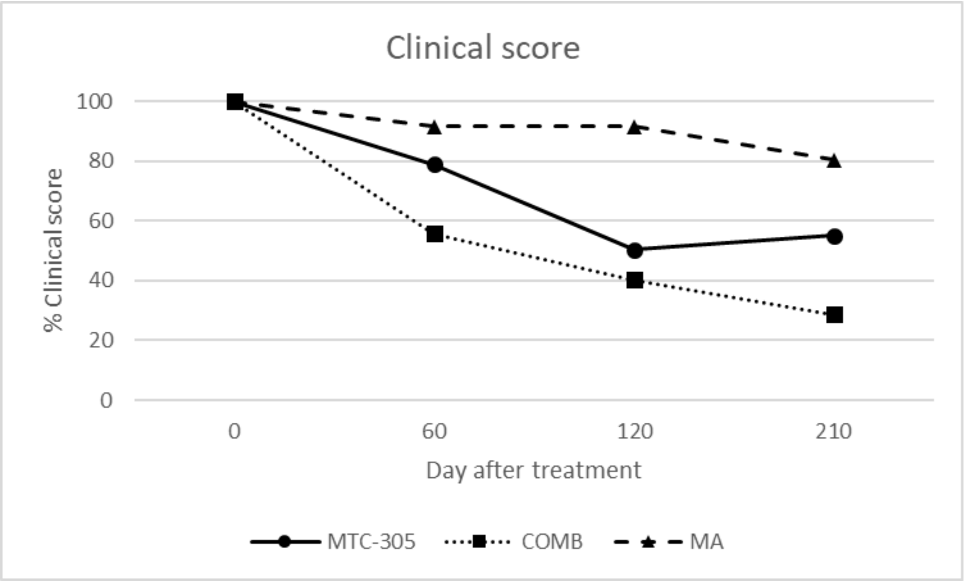

Supplement: Supplementary file 1 [file animals-12-02700-s001.zip › animals-1822017-supplementary.pdf]
